# Signatures associated with homologous recombination deficiency and immune regulation to improve clinical outcomes in patients with lung adenocarcinoma

**DOI:** 10.3389/fonc.2022.854999

**Published:** 2022-09-30

**Authors:** Xueqian Shang, Kang Qi, Xiangzheng Liu, Qinghao Liu, Xining Zhang, Dongliang Wang, Weiming Huang

**Affiliations:** ^1^ Department of Thoracic Surgery, Peking University First Hospital, Beijing, China; ^2^ ChosenMed Technology Co., Ltd., Beijing, China

**Keywords:** LUAD, HRD score, transcriptome, prognostic model, immune

## Abstract

PARP inhibitors can be used to treat solid tumors that often have mutations in important homologous recombination (HR) genes, such as BRCA1/2. While other kinds of tumors could also experience HR deficiencies, including those associated with lung cancer, there is little information on the frequency of these occurrences. Homologous recombination deficiency (HRD) was used to induce particular DNA aberration profiles and related transcriptome alterations. Their presence can identify whether an HR deficiency is present or absent in a particular tumor sample, even without observed HR gene changes. From whole-exome sequencing data in lung adenocarcinoma obtained from TCGA, we obtained several mutational signatures associated with HRD and determined that these HRD-associated mutational signatures are related to genomic installability. We then constructed a prediction model, which found that 11 genes associated with HRD scores could be used as predictors of survival outcomes in LUAD patients. These genes are related to PI3K-Akt, T cell receptors, and the Chemokine pathway. Other GEO datasets validated the survival prediction, which was independent of the PD1/PDL1 treatment. Collectively, our study provides transcriptome biomarkers of lung adenocarcinoma complementary to the HRD score and introduces a novel method of identifying prognostic biomarkers of immunotherapy.

## Introduction

Lung cancer is the most common type of cancer, accounting for 18.4% of worldwide deaths associated with cancer (1). Lung cancer can be grouped into one of two categories: SCLC and NSCLC (of which the most common subgroup is LUAD) (2). Providing a patient with an accurate prognosis can more accurately identify a patient’s risk and guide subsequent treatments, significantly affecting survival rates. One promising new method of cancer treatment includes PARP inhibitors, which work best against tumors with compromised homologous recombination (HR)-mediated DNA repair. PARP inhibitors have been approved to treat ovarian, pancreatic, breast, and prostate cancers, all of which have solid tumors related to loss of function mutations in important HR genes, including BRCA1/2 (1). PARP inhibitor therapy can also benefit other kinds of tumors with HR deficiencies. As such, identifying the incidence of HR deficiencies in these tumor types can significantly benefit clinical outcomes, since they are not often related to germline BRCA1/2 mutations. In 5-10% of NSCLC cases, there are somatic mutations in the BRCA1/2 genes (3, 4); there are also mutations in DNA damage checkpoint genes (5, 6). However, it is unclear whether these mutations can inactivate the HR pathway in patients with lung cancer, though analyzing next-generation sequencing-based DNA aberrations profiles of NSCLC cases can help answer this question.

Loss of function in the HR genes BRCA1 and BRCA2 are related to several mutational characteristics, including (1) a single nucleotide mutational characteristic known as “Signature 3,” the “BRCAness” signature, or SBS3 in COSMIC signatures v3 (7, 8); (2) a mutational profile based on short deletions or insertions which are typically characterized by microhomology deletions and different repair mechanisms joining double-strand breaks when HR is not present (9); (3) large-scale rearrangements, including non-clustered tandem duplications within a particular range of sizes (primarily related to loss of BRCA1 function) or deletions in a 1-10kb range (primarily related to loss of BRCA2 function) (10). Some of these DNA aberration profiles can be specifically induced by loss of function in BRCA1 or BRCA2 in experimental models (9). Composite mutational signatures related to HR deficiency, like the HRD score, can be calculated by assessing the frequency and presence of these mutational events.

Recent studies have demonstrated that gene mutations related to the HR signaling pathway, such as BRCA gene mutations, account for 20% of ovarian cancer patients with HRD (11) (2). As such, additional studies on the traits of transcriptomes in HRD patients can help close these knowledge gaps. While some research has assessed the association between the instability of the tumor genome and the transcriptome (11, 12), there is little information about RNAs associated with HRD or the role they play in lung cancer. Additionally, the BRCA1/BRCA2 genes, which are related to HRD, account for less than 10% of lung adenocarcinoma cases (13). Therefore, additional biomarkers are needed to molecularly type lung adenocarcinoma patients with HRD scores.

We analyzed all available data from the TCGA lung adenocarcinoma (LUAD) cohort and determined which of the above-listed mutational signatures are present in these cases. Based on the HRDscore of the TCGA datasets (3), we estimated the frequency of potential HR deficiency in lung cancer cases. We chose the top 15% and bottom 15% HRD scores differ in their survival rates. We then compared the two group mutation genes and other omics data. Furthermore, we compared epigenetics methylation profiles, transcriptomes (including mRNA and miRNAs), and proteomics. We constructed a geneset prognostic signature model using Cox lasso, which was validated using several independent datasets.

## Methods

### Collection of data

Data on patient multi-omics and related follow-up info were obtained from the publicly available Cancer Genome Atlas (TCGA) database (https://portal.gdc.cancer.gov), while SNV data were obtained from the NCBI Gene Expression Omnibus (GEO) database and the TCGA MC3 groups (4). R was used to normalize the RNA sequencing data as transcripts per million (TPM), while the copy number variation (CNV), number of somatic mutations, fraction genome altered scores (FGA: the ratio of the copy number altered chromosome regions out of the measured regions), and the MSIsensor score (microsatellite instability detection using paired tumor-normal sequence data) were obtained from the TCGA database (3). Data from the GEO samples, including GSE30219 (5), GSE31210 (6), and GSE135222 (7), were used for validation.

We obtained HRD scores from TCGAhrd (https://github.com/GerkeLab/TCGAhrd) (3). Homologous recombination deficiency (HRD) was considered responsible for genomic scarring with large-scale genome instability (14). An HRD score was calculated from the HRD-LOH (8) and LST (large-scale state transitions) (9). **Table S1** displays the HRD scores for each patient.

### Processing of data

To identify other effects, we filtered patients with OS times less than 40 days. Remaining patients were categorized by high HRD scores (n = 74, top 15%) and low HRD scores (n = 70, bottom 15%). Differences in prognostic time were compared using log-rank tests and Kaplan-Meier analysis.

The expression of each gene in the RNA-seq data was normalized using the fragment per kilobase million method and converted according to the Z score. An Illumina Human Methylation 450k array and reverse-phase protein arrays (RPPA) were used for the proteins. Gene expression profiles from the GEO database were processed according to the following: when a gene was mapped to more than one probe, the gene expression was considered the median. We removed probes that were not mapped to a gene ID or that were mapped to more than one gene ID.

We compared the TCGA LUAD data, including mRNA, miRNA, protein, and methylation 450k array. Methylation array analysis was performed *via* ChAMP (10), while other omics were analyzed by DESeq2 (15). RNA-seq data (raw counts) analysis was performed using the “DESeq2” R package. Fold change > 1.5, adj. p< 0.05, were set as the cutoffs to screen for differentially expressed genes (DEGs). The R package cluster profile was used to analyze the functional enrichment of differentially expressed genes and HRD-related DEGs, including GO (11), KEGG (12), and cancer hallmarks (16). We performed a hypergeometric test to assess the significance of our enrichment results; the FDR values were adjusted by GSEA and BH (28).

### Generating risk assessment scores related to HRD scores

We combined different expressions of miRNA, mRNA, methylation, and protein regions as candidate omics genes. We first performed a univariate Cox proportional hazards regression analysis to identify genes significantly associated with OS (P ≤ 0.05). We then established the LASSO Cox regression model using the “glmnet” package (17) and identified genes with beta values that were not zero as possible biomarkers to be used in prognosis. The following formula was used to generate the risk score:

$Riskscore=\sum_{i=1}^{N}Coefficient(Genei)*Expression(Genej)$

**Figure 1:**
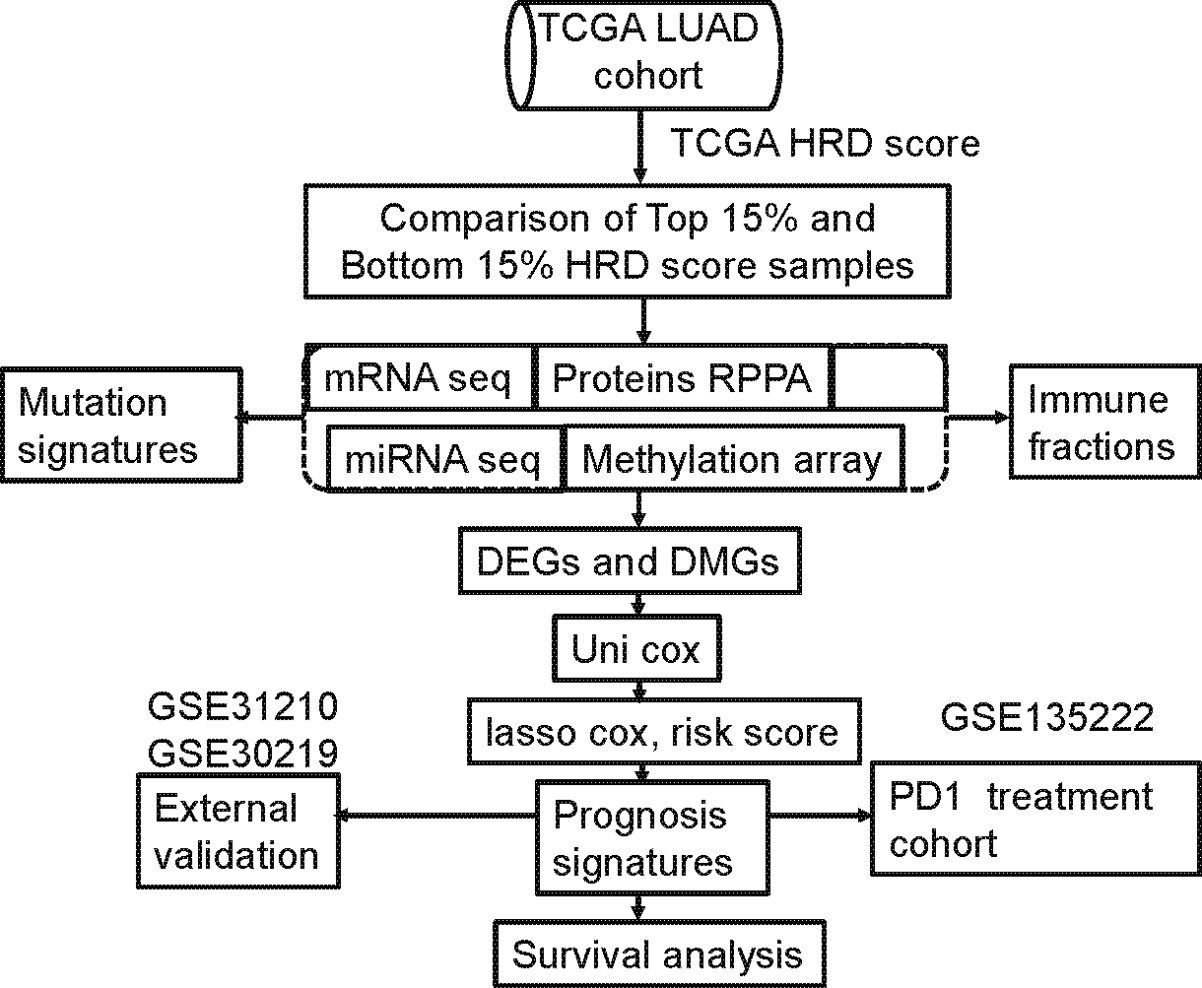
Computational workflow of HRD-related multi-omics detection. HRD-related omics were detected by comparing the RNA expression profile of the highest 15% of patient HRD scores with the bottom 15% of patient HRD scores.

The median risk score was used as a cutoff to categorize patients as low risk (n=234) or high risk (n=233). Differences in prognostic time were assessed using log-rank tests and Kaplan-Meier analyses. **Figure 1** displays the screening process.

### Immune cell infiltration in bulk tumor gene expression data

To study the enrichment of immune cells, we used cibersort (18) and xcell (19), which are efficient algorithms for predicting immune cell infiltration of bulk tumor gene expression data to estimate the abundance of immune cells. We used the CIBERSORT approach to quantify the relative abundance of immune cells using samples from each group, which were then compared between the low and high groups. Additionally, information relevant to immune properties was obtained from the TCGA-LUAD cohort and analyzed between the low and high groups.

### Statistical analysis

We performed Kaplan-Meier survival analysis and the log-rank test using the “survival” R package. Wilcox tests were used to analyze the statistical significance of the differences in two groups, while a Kruskal-Wallis test was used when there were more than two groups. A Mann-Whitney U test was used to analyze the immune cell fraction, TMB, overall survival (OS), and abundance among the low and high groups. The contingency table was analyzed with a Chi-square test and a Fisher’s exact test, while the correlation analysis was performed using Spearman’s correlation coefficient. Survival analysis was performed using the KM method and the log-rank test, where the sirvminer package was used to identify the cutoff ranges for each cohort based on the survival ratio and the ES for each pathway, using maximally selected rank statistics. The level of pathway activation was identified using the median value, after which differences in the number of responders were compared between groups. The hazard ratio was calculated using the univariate Cox proportional hazard regression model, while independent prognostic characteristics were calculated using a multivariate Cox regression model. Results were considered statistically significant when p<0.05, and all statistical tests were two-tailed. We used the ggpubr package (32) to generate the box plots. R software was used to produce all statistical analyses and visualizations (https://www.r-project.org/; version 4.0.0).

## Results

### The instability of each patient’s genomics can be determined with the HRD score, which can be used as a prognostic marker for LUAD patients

**Figure 2:**
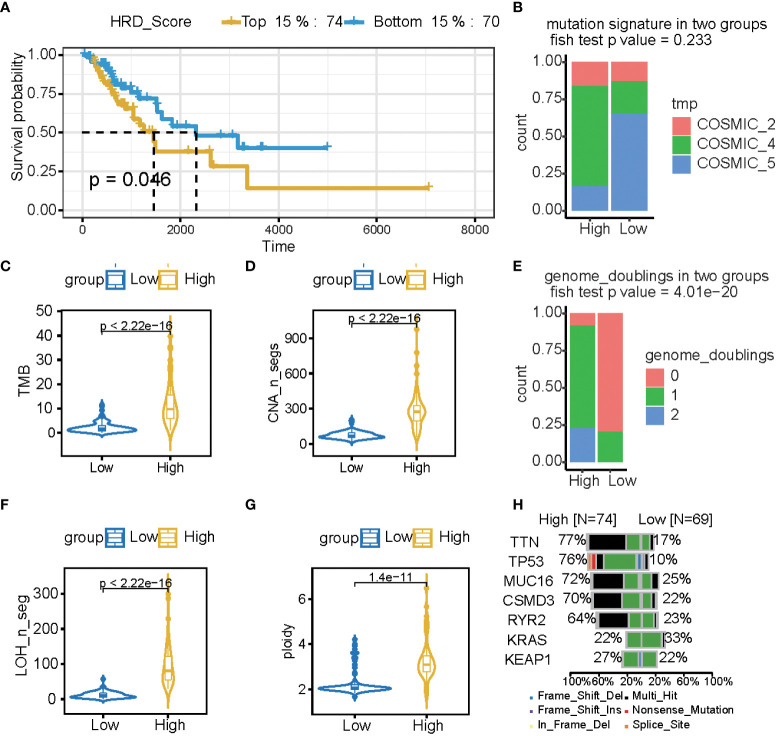
The HRD score reflects a patient’s genomic instability and can be used as a prognostic marker in LUAD patients. **(A)** Kaplan-Meier estimates of overall survival of patients with the top 15% HRD or bottom 15% HRD in the TCGA-LUAD cohort. **(B, E)** Bar plot of mutation signature **(B)** and genome doubling **(D)** in the top 15% HRD-score group and the bottom 15% HRD-score group. **(C, D, F, G)** Violin plot of somatic mutations (TMB), CNA, LOH, ploidy, and subclonal fractions, in the top 15% HRD-score group and the bottom 15% HRD-score group. Somatic mutations in the top 15% HRD-score group were significantly higher than those in the bottom 15% HRD-score group (Wilcoxon signed-rank test). **(H)** Comparison of mutation genes between the top 15% and bottom 15% HRD groups.

LST, TAI, and LOH were used to calculate the HRD score based on the HRD algorithm, while lung cancer patients were classified according to their ascending HRD scores to identify the prognostic capabilities of the HRD score. The bottom 15% and the top 15% of the patients were selected. As shown in **Figure 2A** and **Tables 1**, **S1**, the homologous recombination deficiency (HRD) score was significantly correlated with the prognosis and molecular characteristics of the TCGA-LUAD cohort (p-value = 0.046). We compared the mutation signature and genome doubling with the chi-square test (**Figures 2B, E**) and found that it differs for these two groups. The high group is enriched with cosmic signature 4, which has a similar mutational pattern seen in experiments dealing with tobacco carcinogens (e.g., benzo[a]pyrene) and is related to smoking. Tobacco mutagens are likely responsible for signature 4, while the low group is enriched with cosmic signature 5. The high group experienced more genome doubling, and the low group experienced less genome doubling (**Figure 2E**). We subsequently investigated the correlation between the HRD score and other characteristics of genomic instability, including somatic mutation counts for TMB, CNA, LOH, ploidy, and subclone fractions. The median value of somatic cumulative mutations in the HRD group was significantly higher in the HRD high group than in the HRD low group (Wilcoxon signed-rank test, p<.0001) (**Figures 2C, D, F, G** and **Supplementary Figure 1**). This indicates that the genome mutation profiles differ between these two groups. Lastly, we compared the mutation genes between these two groups and found that TTN, TP53, MUC16, CSMD3, RTR2, and KEAP1 have higher mutation rates in the high group, while KRAS is higher in the low group (**Figure 2H**).

**Table 1:** The characteristics of samples in the TCGA-LUAD dataset.

Variant	High	Low	p
	74	70	
FEMALE	32 (43.2)	39 (55.7)	0.184
MALE	42 (56.8)	31 (44.3)	
age	62.18 (10.43)	66.88 (9.43)	0.006
Former smoker for< or = 15 years	29 (39.2)	16 (23.9)	<0.001
Former smoker for > 15 years	11 (14.9)	24 (35.8)	
Former Smoker, Duration Not Specified	0 (0.0)	1 (1.5)	
Current smoker	32 (43.2)	13 (19.4)	
Lifelong Non-smoker	2 (2.7)	13 (19.4)	
Stage I	38 (51.4)	43 (62.3)	0.470
Stage II	23 (31.1)	16 (23.2)	
Stage III	12 (16.2)	8 (11.6)	
Stage IV	1 (1.4)	2 (2.9)	
HRD TAI	20.91 (4.02)	2.74 (1.73)	<0.001
HRD LST	18.12 (5.29)	1.66 (1.21)	<0.001
HRD LOH	12.32 (3.92)	1.61 (1.23)	<0.001
HRD Score	51.35 (8.52)	6.01 (3.20)	<0.001

### HR deficiency-associated DEGs in lung adenocarcinoma patients

**Figure 3:**
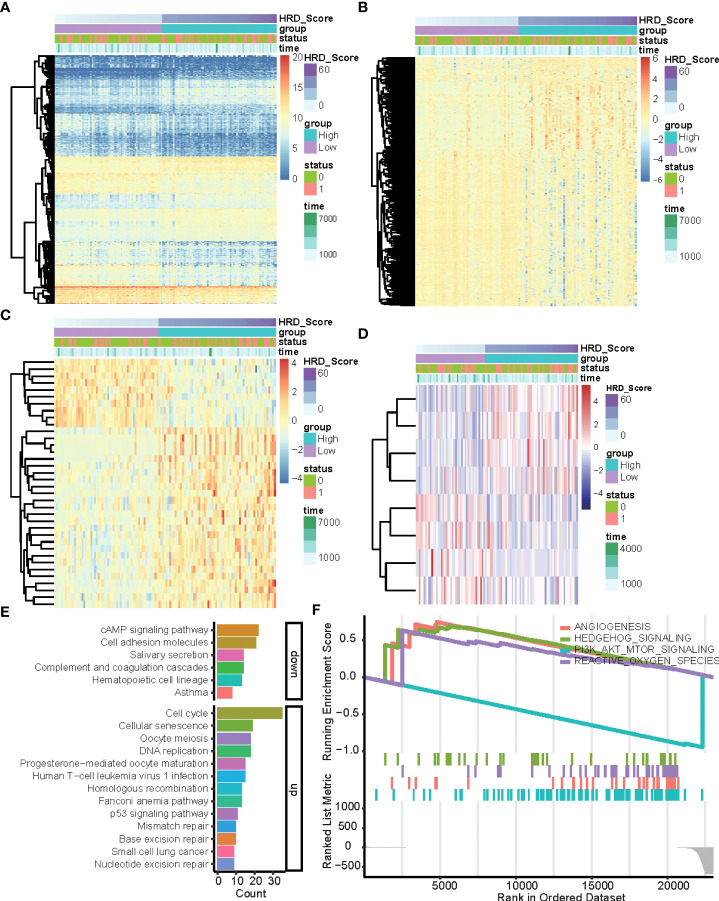
DEGs and related pathways associated with HRD. **(A–D)** Heatmap of DEGs in the top 15% HRD-score group and the bottom 15% HRD-score group. **(E)** KEGG enrichment analysis of DEGs. The heights of the columns indicate DEG counts in the total number of genes in the signaling pathway and the color depth represents the pathways. **(F)** GSEA Enrichment plot (cancer hallmark pathways) in the top 15% HRD-score group and the bottom 15% HRD-score group.

To explore the multi-omics signatures associated with HRD, we compared the difference of the whole transcriptome between the top 15% HRD-score group and the bottom 15% HRD-score group (**Figure 1**). Utilizing the DESeq2 method, a total of 494 different methylation sites, 1,348 differentially expressed genes (DEGs), 36 DE miRNAs, and 9 RPPA differentially expressed proteins were screened out in lung cancer patients (**Figures 3A–D**, **Supplementary Figures 3**, **4** and **Table S2**). KEGG and GO cluster plots revealed that immune-related signaling pathways, including human T−cell leukemia virus 1 infection and DNA repair pathways, the p53 signaling pathway, homologous recombination, mismatch repair, and nucleotide excision repair were observed in the top 15% HRD-score group, while cAMP signaling pathway and cell adhesion molecules are enriched pathways in the bottom 15% HRD-score group (**Figure 3E**). Furthermore, in the hallmark GSEA result, the PI3K AKT mTOR is downregulated, while angiogenesis and reactive oxygen are upregulated in the top 15% groups (**Figure 3F**).

### Establishment of risk assessment signature

**Figure 4:**
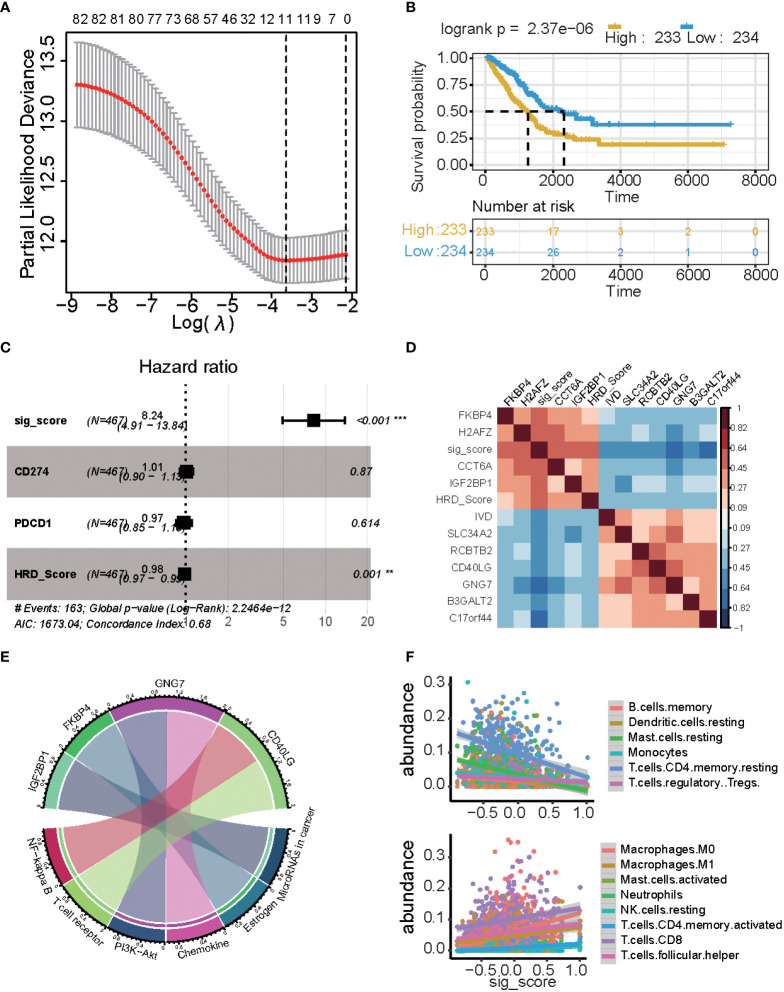
Results of the prognostic signature model. **(A)** The correlation between log(λ) and deviance. **(B)** Kaplan-Meier estimates of overall survival of patients with high- or low-risk scores in the TCGA-LUAD cohort. **(C)** Multivariate Cox regression analysis of the signature with PD1/PDL1 and HRDscore were taken into account (*P ≤ 0.05, **P ≤ 0.01, ***P ≤ 0.001). **(D)** Heatmap displaying the relationship between the signature genes. **(E)** Chord plot depicting the relationship between genes and immune-related signaling pathways. Genes marked in red fonts refer to the most frequently repeated genes in immune-related signaling pathways. **(F)** Scatterplot displaying the signature score with immune cell fractions, including positively related and negatively related.

A total of 1,348 genes, 28 miRNAs, 8 proteins, and 79 methylation sites were integrated into a gene set, which was subjected to a univariate Cox regression analysis. 241 potential candidate genes were pinpointed (P ≤ 0.05), including 85 markers. In LASSO regression, the optimal λ value pointed to the most robust prognosis signatures, after 10-fold cross-validation with 1000 repeats. The remaining 11 genes had non-zero LASSO coefficients, including B3GALT2, C17orf44, CCT6A, CD40LG, FKBP4, GNG7, H2AFZ, IGF2BP1, IVD, RCBTB2, and SLC34A2 (**Figure 4A**). Their corresponding LASSO coefficients are displayed in **Figure 4** (**Supplementary Table S3**). Finally, the risk score was calculated using the formula in the “Construction of Risk Assessment Signature” section.

There was a significant difference in the survival rate between the low-risk group and the high-risk group (P = 2.37e−06) (**Figure 4B**). We used multi-cox analysis to analyze the PD1, PDL1, HRD score, and signature score and found that the signature score p-value< 0.05 (**Figure 4C**). For these signature genes, FKBP4, H2AFZ, CCT6A, and IGF2BP1 are positively related to the signature score, while the HRD score, IVD, SLC34A2, RCBTB2, CD40LG, GNG7, B3GALT2, and C17orf44 are negatively related to signature and HRD score (**Figure 4D**).

These genes are related to the immune system and tumors: GNG7 is related to PI3K-Akt and Chemokine, FKBP4 is related to Estrogen, and IGF2BP1 is related to miRNA in the cancer pathway, and CD40LG is related to the NF-kappa B and T cell receptors (**Figure 4E**). There is a significant correlation between the signature score and cibersort cell abundance. B memory cells, resting dendritic cells, resting mast cells, monocytes, and resting memory CD4 T cells are positively associated with the signature score (p< 0.05), while regulatory T cells are negatively associated with the signature score. Macrophages, M0 cells, Macrophages M1 cells, activated mast cells, neutrophils, resting NK cells, activated memory CD4 T cells, CD8 T cells, and follicular helper T cells are positively associated with signature scores (p< 0.05).

### Validation in other lung cancer datasets

**Figure 5:**
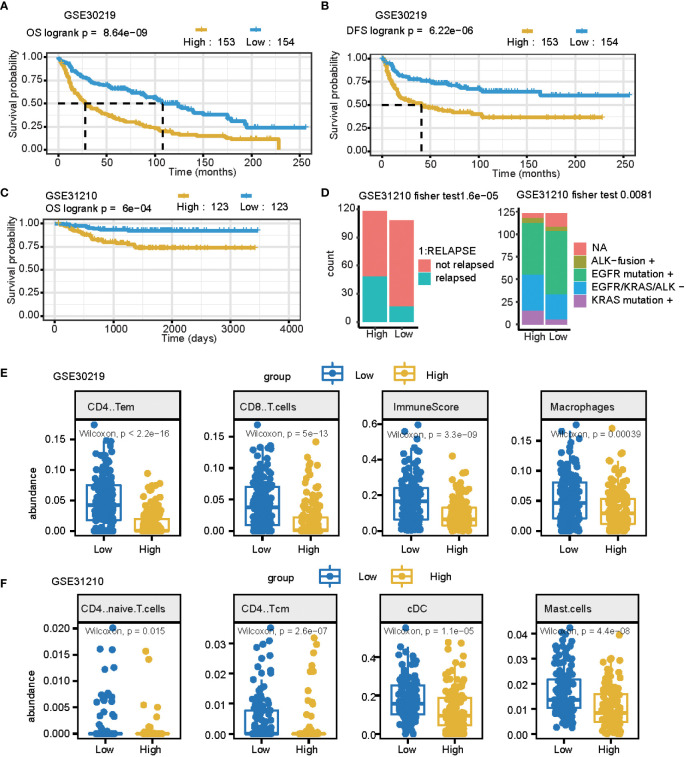
The prognostic signature can be used as a potential biomarker for LUAD. **(A, B)** Curve for OS (overall survival) **(A)** and DFS (disease-free survival) **(B)** are shown for the high and low signature scores in lung cancer. **(C)** The curve for OS (overall survival) is shown for the high and low signature scores in lung adenocarcinomas. **(D)** Summary of clinical and mutation burden differences across the two groups for the high and low signature scores in lung cancer. Testing differences of corresponding P-values in relapse and mutation genes. **(E)** Summary of differences in immune cell fractions between the two groups for the high and low signature scores in the lung cancer GSE30219 dataset. This includes CD4 T memory cells, CD8 T cells, macrophages, and immune scores. **(F)** Summary of differences of immune cell fractions between the two groups for a high and low signature score in the lung cancer GSE31210 dataset. This includes CD4 naïve T cells, CD4 central memory cells, classical dendritic cells, and mast cells.

An independent external cohort was used to confirm the prognostic efficacy of the 11 gene signatures. Similarly, the OS and RFS of patients with higher risk scores are significantly worse than that of patients with lower risk scores (p = 0.0011, **Figures 5A, B** GSE30219 datasets). In the GSE31210 dataset, the high-risk group had more relapsed samples (Fisher’s exact test, p< 0.001) (**Figure 5C**). The relapsed status was different: the high signature score groups had more relapsed patients and the mutation genes differed between these two groups (Fisher’s exact test, p< 0.01) (**Figure 5D**). Furthermore, we compared the cell fraction between these two groups and found several different immune cells in the high and low signature score groups. CD4 T memory cells, CD8 T cells, macrophages, and immune scores were all significantly higher in the low group in the GSE30219 datasets (**Figure 5E**). CD4 naïve T cells, CD4 central memory cells, classical dendritic cells, and mast cells were all higher in low groups in GSE31210 datasets (**Figure 5F**).

### Comparison of signature scores with immune therapy PD1 drugs

**Figure 6:**
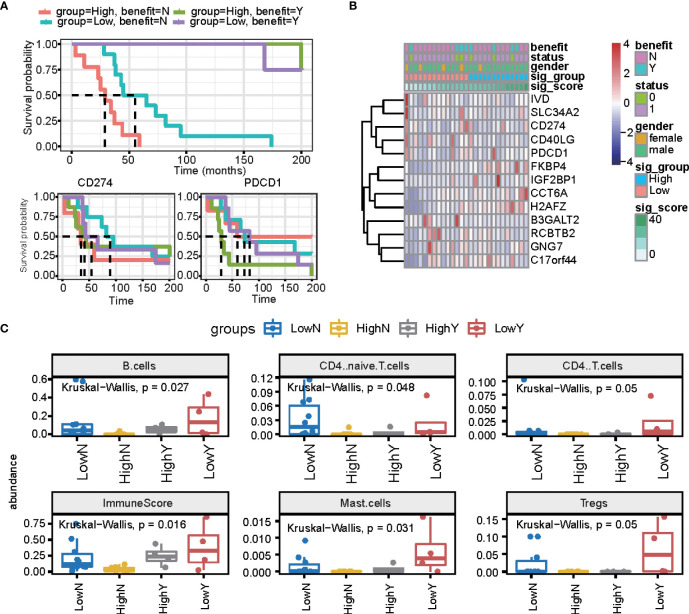
The prognostic signature is independent of PD1/PDL1 treatment. **(A)** The overall survival curve is shown for high and low signature scores in the PD-L1 treatment cohort. **(B)** Heatmap displaying the expression of signature genes and PD1/PDL1. **(C)** Summary of immune cell fractions differences across the 4 groups. LowN, low signature scores, and no benefit; HighN, high signature scores, and no benefit; HighY, high signature scores, and benefit; LowY, low signature scores, and no benefit. This includes B cells, CD4 naïve cells, CD4 T cells, immune score, mast cells, and regulated T cells.

We further estimated the signature score in PD1/PDL1 treatment patients (GSE135222 dataset, which is PD1/PDL1 treatment patients), and we grouped patients by drug response. This means that there are no-response patients in “no benefit groups” and response patients in “benefit groups.” For the response groups, the OS was better, and was considered the benefit group; in the no-benefit samples (the no-benefit group), we grouped patients by the median of their signature scores into high signature score groups and low signature score groups. The high-risk group was worse than the low-risk group (**Figures 6A, B**). Under PD1/PDL1 treatment, the gene expression of PDCD1 (PD1) and CD274 (PDL1) had no significant different survival prognostics for PD1/PDL1 expression, and the signature score high and low groups (**Figure 6A**). We then compared the fractions of other immune cells between the four groups (LowN, low signature scores, and no benefit; HighN, high signature scores, and no benefit; HighY, high signature scores, and benefit; LowY, low signature scores, and no benefit.). There were more B cells, CD4 T cells, mast cells, and regulatory T cells in the signature score low and benefit groups. The immune score was relatively high for the high signature score and benefit groups (**Figure 6C**).

**Figure 7:**
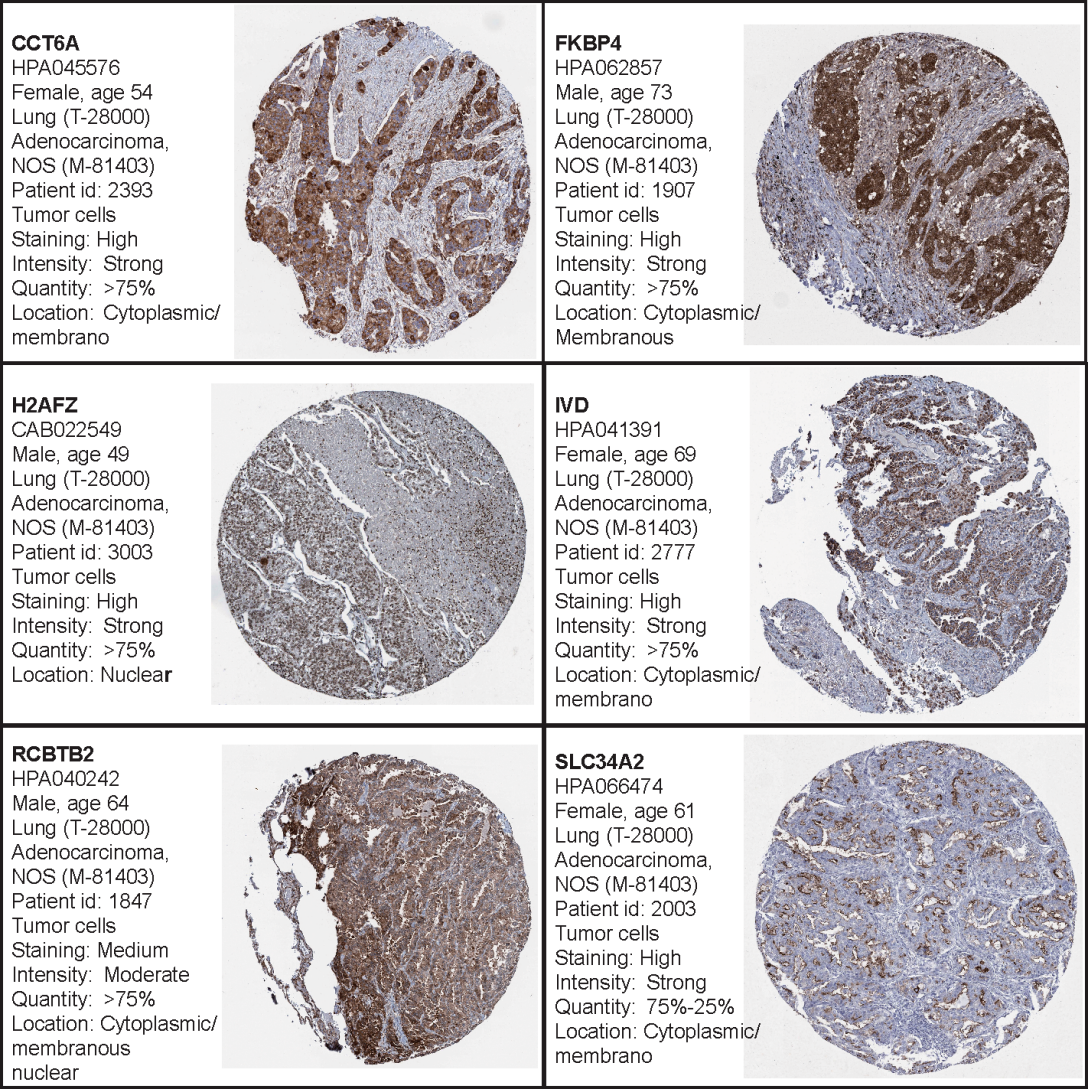
The Human Protein Atlas (HPA) database was used to validate the four hub genes. No data for B3GALT2, C17orf44, CD40LG, GNG7, or IGF2BP1 were available in the HPA database.

Additionally, the Human Protein Atlas database was used to obtain the hub genes, while IHC was used to determine their expression. Our results indicate that expression levels were related to transcription levels, though there was no IHC data available for B3GALT2, C17orf44, CD40LG, GNG7, and IGF2BP1 (**Figure 7**).

## Discussion

In this study, we analyzed the molecular characteristics of LUAD patients with different HRD scores and identified biomarkers that can be used to complement the HRD score. A comprehensive analysis of the genomic landscape of the HRD score group demonstrated a high abundance of TMBs and a difference in genome doublings, and mutation signatures. We performed univariate Cox proportional hazards analysis and LASSO COX regression analysis to screen multi-omics genes associated with the HRD score prognosis. The prediction model was established using 11 mRNA genes. Our study provides a robust model and candidate biomarkers for personalized therapy of LUAD patients. The 11 gene signature model is a potential reliable biomarker for assessing prognosis, while the predictive effect of the signature model was independent of PD-1/PD-L1.

While lung cancer is not typically related to germline BRCA1/2 mutations, there have been a few instances (32, 33): there are somatic in the BRCA1 or BRCA2 gene in approximately 5-10% of non-small cell lung cancer cases (4). We determined that HRD scores are suitable for use as a biomarker when complementing the mutation status of genes associated with HR. We observed higher rates of cosmic mutation signature 4 in the high HRD score group, which is associated with tobacco. Additionally, we observed more genome doubling in the high group. The TMB, CNA, LOH, and ploidy were all high in the high group, which indicates the presence of additional genome-wide mutations and alternations and could introduce additional tumor antigens to induce an immune response. These genes include TTN, TP53, MUC16, CSMD3, RYR2, KRAS, and KEAP1, while TP53 had higher mutation rates in the high group, and KRAS had higher mutation rates in the low group. There could be different evolutionary pathways for these two groups, though KRAS is significantly mutually exclusive with TP53 (20).

We performed a multi-omics comparison of the high and low groups, epigenetics, transcriptome, and proteomics, and found thousands of differentially expressed features in various methylation sites. These genes are involved in DNA repair-related pathways in the high HRD score group, which is consistent with genome-level modifications. In these cases, the high HRD score activates the DNA repair and immune response. The 11 signature genes are associated with the immune pathways NF-kappa B, T cell receptor, and chemokine. Regulation of the immune system can be further validated when estimating immune cell abundance, which is similar to the HRD score and immune checkpoint blockade (14, 21).

Furthermore, the signature score of 11 genes is unrelated to the PD1/PDL1 treatment. There were no significant differences in prognosis for PD1/PDL1 benefit patients, while there were significant differences in prognosis for PD1/PDL1 no benefit patients. Previously, we assessed the difference between PD1/PDL1 expression with the signature and found that it is independent of PD1/PDL1. However, these genes are associated with various immune regulatory pathways, meaning that these genes could be changing immune-regulatory pathways and could represent potential targets for immune therapy.

Our study had a few limitations. There was no transcriptomic data of LUAD with PARP inhibitors treatment, which limited our direct validation of 11 genes. Therefore, we used CRISPR/Cas9 and pharmacodynamic data to assess the ability of the 11 genes to predict the HRD population and its susceptibility to PARP inhibitors. Additionally, we were unable to identify the co-inhibition effect with PARP inhibitors using public data since there are few studies assessing HJURP and CDCA2 inhibitors. These results were produced by analyzing bioinformatics and would benefit from additional clinical verification of the 11 genes and the validation of using the cell cycle pathway and PARP co-inhibition in animal and cell line experimental models.

In conclusion, this study provides a novel perspective on the molecular traits of the genomics and transcriptomes of LUAD patients. We determined that HRD scores can be used as prognostic biomarkers in LUAD patients. Additionally, we found that the 11 gene expression signature model can predict the survival outcome of LUAD patients and could serve as a potential biomarker for assessing the effectiveness of immune signatures. This study possesses high value for applications in clinical settings and lays the groundwork for onboarding LUAD patients in precision medicine programs.

## Data availability statement

The original contributions presented in the study are included in the article/**Supplementary Material**. Further inquiries can be directed to the corresponding author.

## Author contributions

XS and WH designed the study; XS, KQ, and XL performed the experiments, XS, DW, and XZ analyzed the data; XS, DW, and WH wrote the manuscript. All authors contributed to the article and approved the submitted version.

## Acknowledgments

This work was supported by the Youth clinical research project of Peking University First Hospital 2019CR07.

## Conflict of interest

Author DW is employed by ChosenMed Technology Co., Ltd., Beijing.

The remaining authors declare that the research was conducted in the absence of any commercial or financial relationships that could be construed as a potential conflict of interest.

## Publisher’s note

All claims expressed in this article are solely those of the authors and do not necessarily represent those of their affiliated organizations, or those of the publisher, the editors and the reviewers. Any product that may be evaluated in this article, or claim that may be made by its manufacturer, is not guaranteed or endorsed by the publisher.
