# Supplementary figures and images for: Signatures associated with homologous recombination deficiency and immune regulation to improve clinical outcomes in patients with lung adenocarcinoma
Source: Front Oncol. 2022 Sep 30;12:854999. doi: 10.3389/fonc.2022.854999 (PMC9562462; doi:10.3389/fonc.2022.854999)

group Low High

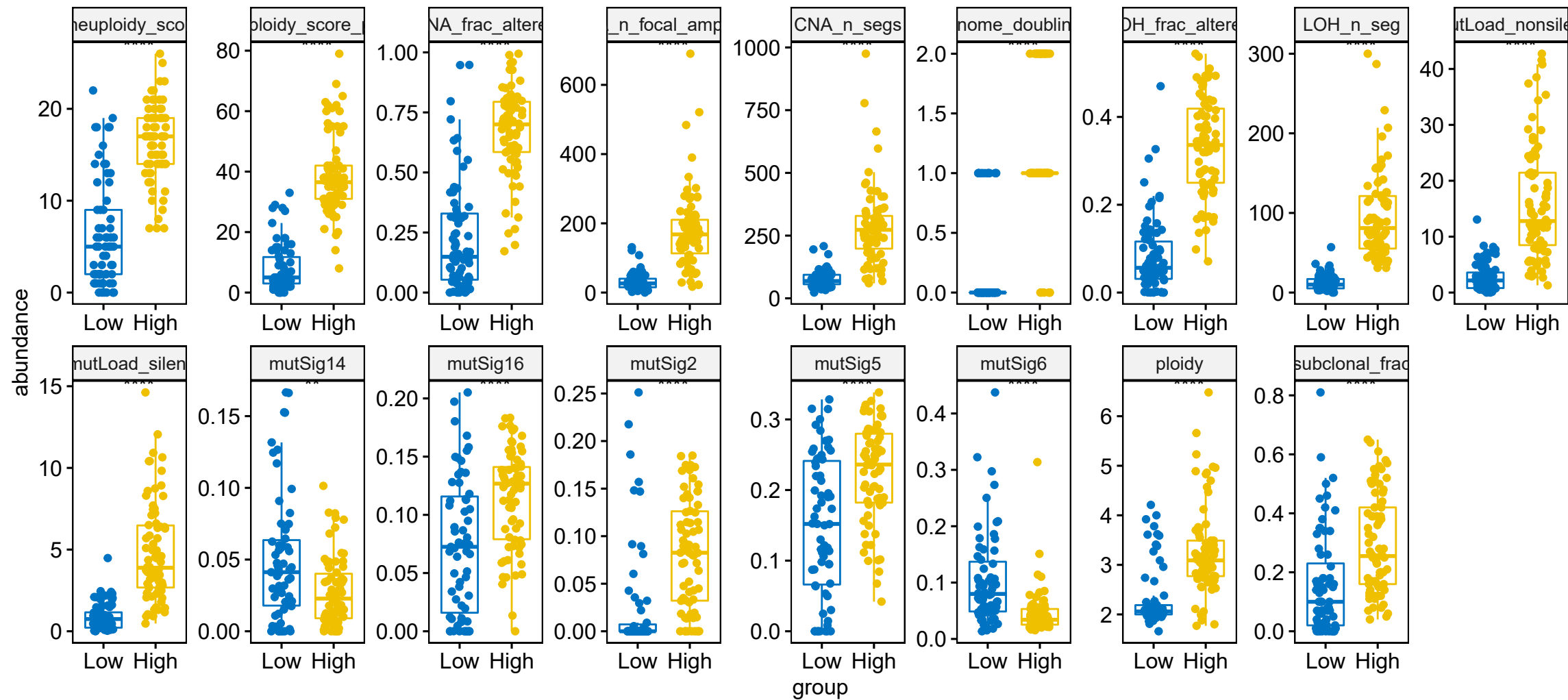

Supplement: Supplementary file 4 [file Image_1.pdf]

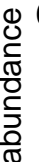

Supplement: Supplementary file 6 [file Image_3.pdf]

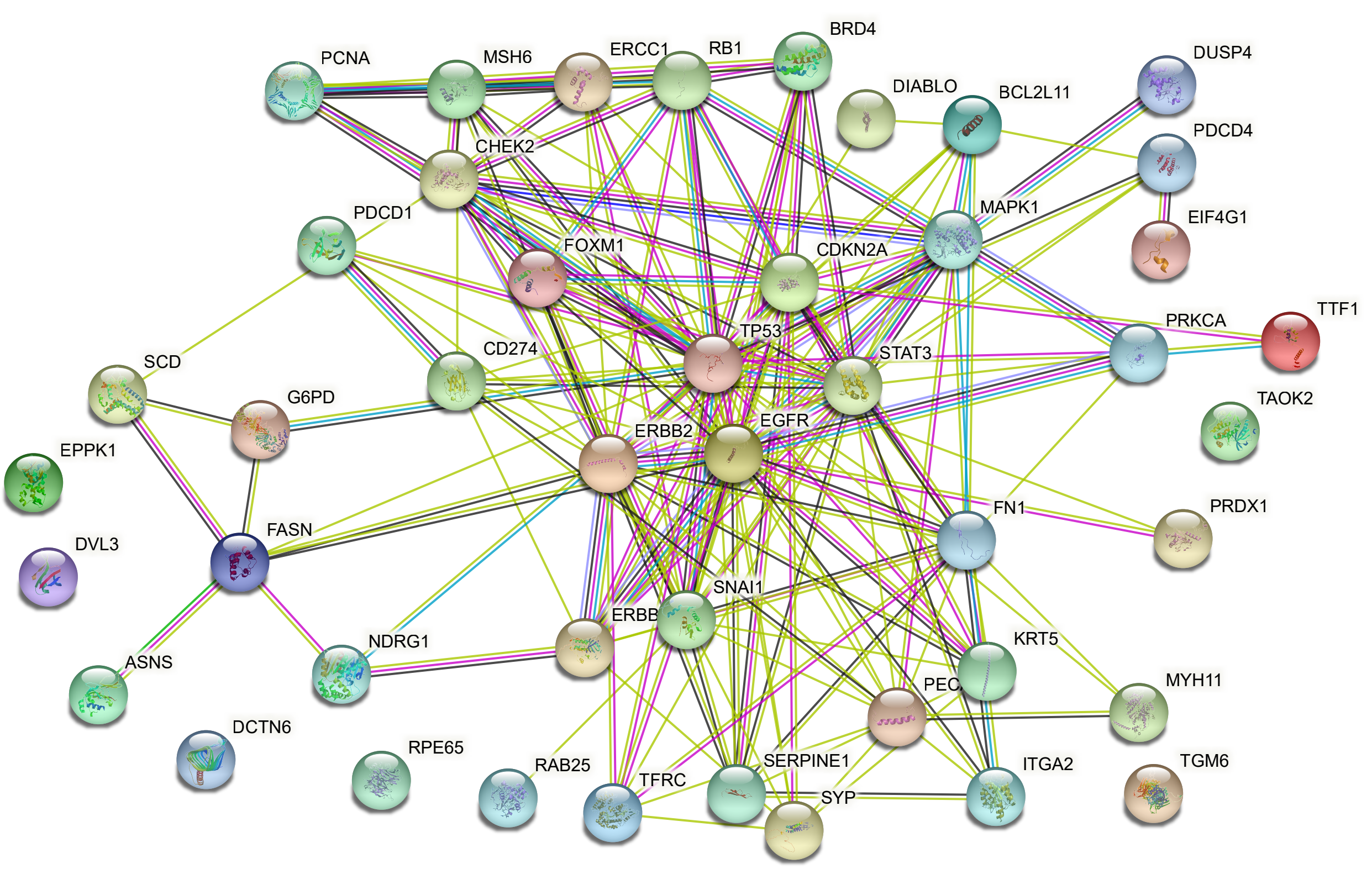

Supplement: Supplementary file 7 [file Image_4.pdf]
