# Supplementary material for: Signatures associated with homologous recombination deficiency and immune regulation to improve clinical outcomes in patients with lung adenocarcinoma
Source: Front Oncol. 2022 Sep 30;12:854999. doi: 10.3389/fonc.2022.854999 (PMC9562462; doi:10.3389/fonc.2022.854999)
Supplement: Supplementary file 5 [file Image_2.pdf]

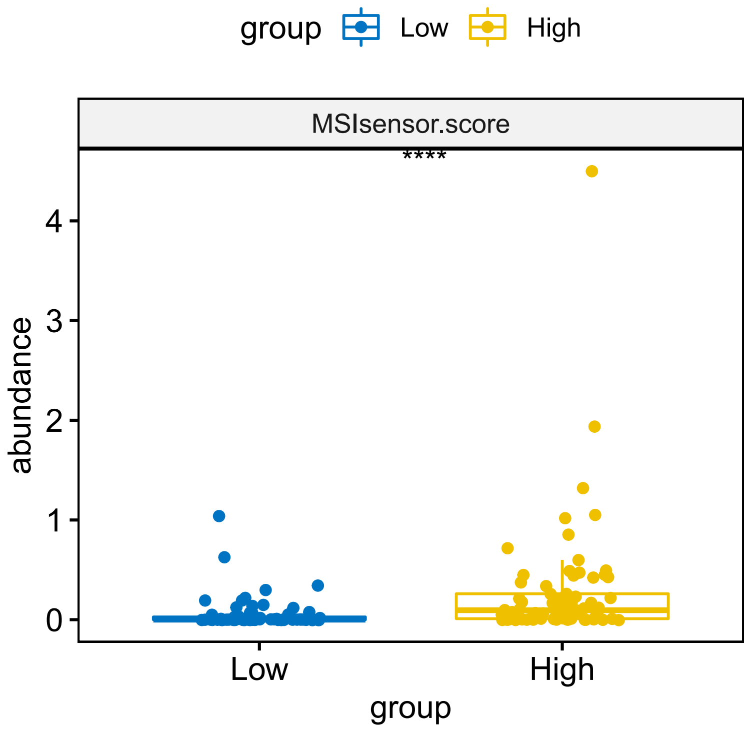
group
Low
High
MSIsensor.score
****
abundance
0
1
2
3
4
Low
High
group
